# Efficient Separation of Methanol Single-Micron Droplets by Tailing Phenomenon Using a PDMS Microfluidic Device

**DOI:** 10.3390/molecules29091949

**Published:** 2024-04-24

**Authors:** Daiki Tanaka, Shengqi Zheng, Masahiro Furuya, Masashi Kobayashi, Hiroyuki Fujita, Takashiro Akitsu, Tetsushi Sekiguchi, Shuichi Shoji

**Affiliations:** 1 Department of Electronic and Physical Systems, School of Fundamental Science and Engineering, Waseda University, Tokyo 145-0065, Japan; zhengshengqi@fuji.waseda.jp (S.Z.); shojis@waseda.jp (S.S.); 2 Cooperative Major in Nuclear Energy, Waseda University, Tokyo 169-8555, Japan; mfuruya@waseda.jp (M.F.); maajii@fuji.waseda.jp (M.K.); 3 Canon Medical Systems Corporation, Otawara 324-8550, Japan; hfujita@iis.u-tokyo.ac.jp; 4 Department of Chemistry, Faculty of Science, Tokyo University of Science, Tokyo 162-0825, Japan; akitsu@rs.kagu.tus.ac.jp; 5 Research Organization for Nano & Life Innovation, Waseda University, Tokyo 162-0041, Japan; t.sekiguchi@ruri.waseda.jp

**Keywords:** organic microdroplets, single-micron droplets, fluid control, surface treatment

## Abstract

Microdroplet-based fluidic systems have the advantages of small size, short diffusion time, and no cross-contamination; consequently, droplets often provide a fast and precise reaction environment as well as an analytical environment for individual molecules. In order to handle diverse reactions, we developed a method to create organic single-micron droplets (S-MDs) smaller than 5 μm in diameter dispersed in silicone oil without surfactant. The S-MD generation microflow device consists of a mother droplet (MoD) generator and a tapered separation channel featuring multiple side channels. The tapered channel enhanced the shear forces to form tails from the MoDs, causing them to break up. Surface treatment with the fluoropolymer CYTOP protected PDMS fluid devices from organic fluids. The tailing separation of methanol droplets was accomplished without the use of surfactants. The generation of tiny organic droplets may offer new insights into chemical separation and help study the scaling effects of various chemical reactions.

## 1. Introduction

Microfluidics involves manipulating continuously flowing microscopic amounts of fluids within channels of nanometer/micron dimensions. This technology originated in the 1950s when Skeggs proposed an automated approach for colorimetric analysis by controlling fluid flow [1], followed in 1998 by a paper by Xia and Whitesides [2] on a polydimethylsiloxane (PDMS) soft lithography method. The surface–volume ratio of a fluid in a microfluidic device gradually increases as the fluid volume decreases, which leads to properties distinct from those of macroscopic fluids. Microfluids exhibit three main characteristics: efficient mass/heat transfer, sufficient viscosity for overcoming inertial forces, and significant surface effects [3,4,5]. These features make it possible to control and manipulate individual fluids and fluid interfaces using miniaturized devices, enabling the application of microfluidics in areas such as physics, chemistry, biology, medicine, and engineering [6,7,8]. In 2001, Thorsen et al. achieved droplet shearing, enabling the successful development of droplet microfluidic devices [9]. A new branch of discontinuous-flow microfluidic systems, known as droplet microfluidic systems, uses two immiscible liquids to form droplets on the micron or nanometer scale at the flow interface. Recent advances in droplet preparation and manipulation techniques allow for the sorting [10], merging [11], splitting [12], mixing [13], and capturing [14] of droplets inside microfluidic devices. These technologies have led to dramatic advances in cell culture technology in droplets [15,16,17,18]. Joachim D. J. and colleagues reported that concentrating the droplets improved gene detection rates by five times and reduced background noise by half [19]. Wang et al. [20] reported that a microdroplet-based surface-enhanced Raman spectroscopy (microdroplet SERS) platform was constructed to envelop individual cells with microdroplets, followed by droplet immunization using immunomagnetic beads. Extracellular vesicle proteins (EV proteins) were detected by SERS assay [20]. Single-cell analysis is expected to be further developed with technology that stably generates single-micrometer droplets. Microdroplets have also been applied in the field of chemistry [21,22,23]. Ashleigh B. and colleagues used microfluidic technology and a new fluorous-tagged palladium catalyst to generate droplet reactors with catalytically active walls and used these compartments for small molecule synthesis [24]. Tim-A. M. et al. reported analyzing the progress of reactions in situ through the surface-enhanced Raman spectroscopic monitoring of fast-moving individual droplets [25]. In recent years, the analysis of microdroplet generation and agitation has been performed using CFD simulation and image analysis [26,27,28]. M. Rahimi et al. reported a detailed analysis and discussion of droplet generation related to the Co-axial Flow Focusing based on simulation and image analysis [29].

Droplet separation and recovery devices mainly utilize multilayer flow extraction and separation to isolate the target products [30]. So far, we have succeeded in generating single-micron droplets from approximately 100 μm droplets using water and oil by applying the tailing phenomenon [31]. Most experiments to date have been conducted on an aqueous phase dispersed in an oil phase. Organic solvents play crucial roles in organic reactions, but there have been few studies on the application of relevant organic droplets.

Here, we generated organic droplets less than 5 μm in diameter to provide a suitable environment for chemical reactions at the nanoscale. However, it was necessary to address two critical problems. The first was the incompatibility of organic solvents with conventional microfluidic devices fabricated by soft micro-electromechanical system processes using PDMS because PDMS tends to swell upon contact with organic solvents, leading to the deformation of the flow path. The second was the typical need for surfactant addition to generate tiny droplets because surfactants can react electrostatically with substrate molecules, which results in reactions that interfere with the target reaction [32].

We are interested in studying the scaling effects of chemical reactions. We thus aimed to generate organic droplets less than 5 μm in diameter without the use of surfactants. Here, we chose methanol, which has a low interfacial tension, as the organic solvent for droplet generation, and silicone oil as the continuous phase to stabilize the generated droplets. Furthermore, we intended to use a PDMS device to generate organic droplets over an extended period and to reduce the affinity of organic solvents for the inner walls of the flow channel. To this end, we surface-treated the inner walls with the fluoropolymer CYTOP (CTL-809M, AGC Inc., Tokyo, Japan) to alter the surface wettability. Finally, organic droplets less than 5 μm in diameter were successfully separated from the parent droplets by configuring the flow channel and fluid pressure.

## 2. Results and Discussion

### 2.1. Fluid Simulation (CYTOP Surface Treatment)

**Figure 1:**
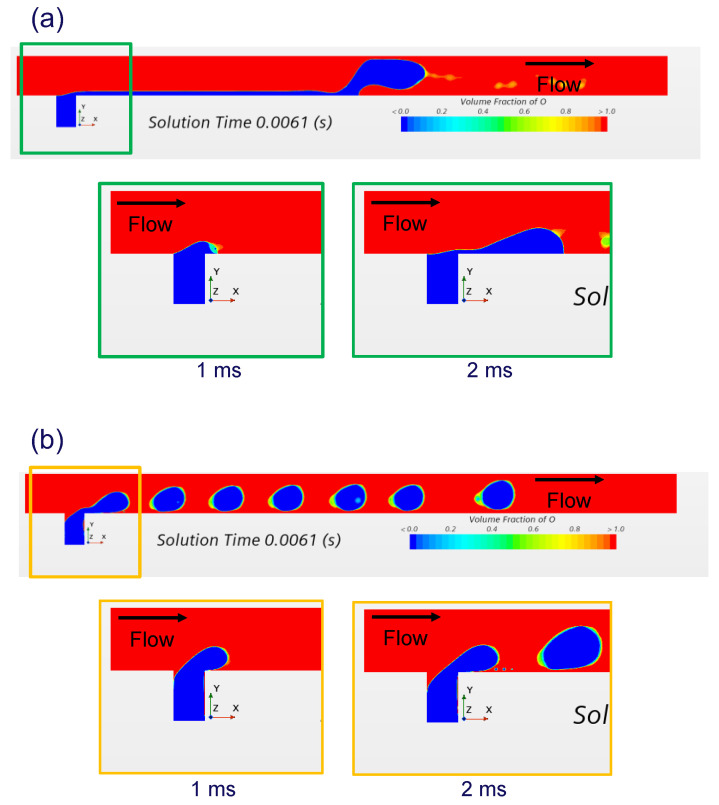
Scenes of three-dimensional CFD simulation in T-junction [28]: (**a**) Contact angle of 30 degrees. (**b**) Contact angle of 150 degrees.

Figure 1 shows scenes of the three-dimensional CFD simulation in a T-junction. The contact angle of the methanol to the channel wall was changed. The methanol in blue ink is injected in the mainstream of the silicone oil in red ink. Figure 1a shows CFD scenes for a contact angle of 30 degrees (which simulates on the PDMS wall). The methanol is so wettable that the methanol stream creeps on the PDMS wall. Figure 1b shows CFD scenes for a contact angle of 150 degrees. Since the methanol is less wettable (which simulates on the CYTOP wall), the methanol stream takes off from the wall in the T-junction. After that, the disintegrated ethanol stream generates a series of droplets. The CFD simulation confirms the role of wettability (by coating of CYTOP) to generate a series of droplets.

### 2.2. Fluid Experiments

**Figure 2:**
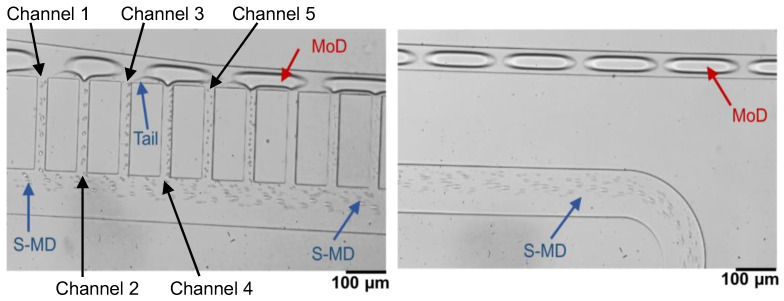
Optical microscopy images showing S-MD formation.

The S-MD separation performance of the device was evaluated by optical microscopy observations at the droplet separation section of the branched channel. Figure 2 illustrates an example of such separation, where the flow rates of the methanol and oil are 1.2 μL/min and 4.5 μL/min, respectively. Several MoDs can be observed in the main channel at the top of the image. After entering the side channel section, the deformed MoDs experience pressure in the direction of the side channels and shear forces from the channel corners, leading to the generation of a tail and its separation into the S-MDs. As observed in Figure 2, the MoDs are subjected to forces from the flow into the up-stream side channel to form a tail. The S-MDs are separated during the continuous motion owing to the differences in velocity and the direction of the motion of the MoDs and the tails. The separated S-MDs are then extracted into the downstream side channel. Thus, the fabricated device allowed the continuous generation of the S-MDs efficiently.

### 2.3. Relationship between Continuous-Phase Flow Rate and S-MD Diameter

To investigate the relationship between the flow rate of the continuous phase (silicone oil) and the diameter of the generated S-MDs, the flow rate of the dispersed phase (methanol) was fixed at 1.5 μL/min, and the flow rate of the continuous phase was set to 3, 4, 5, or 6 μL/min.

**Figure 3:**
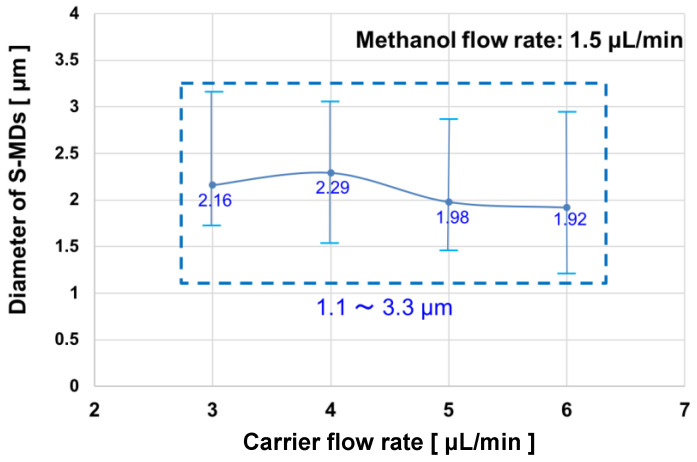
Methanol S-MD diameter under different carrier flow rates.

The diameters of the S-MDs generated at the fifth side channel are plotted against the flow rate in Figure 3. The S-MDs of 1.1–3.3 μm were formed under all flow conditions.

### 2.4. Drop Diameter Distribution Based on Image Analysis

Image analysis was conducted for the fluid experiment movies at the continuous flow rates of 3 μL/min, 4 μL/min, 5 μL/min, and 6 μL/min, and for fixed dispersed flow at 1.5 μL/min.

**Figure 4:**
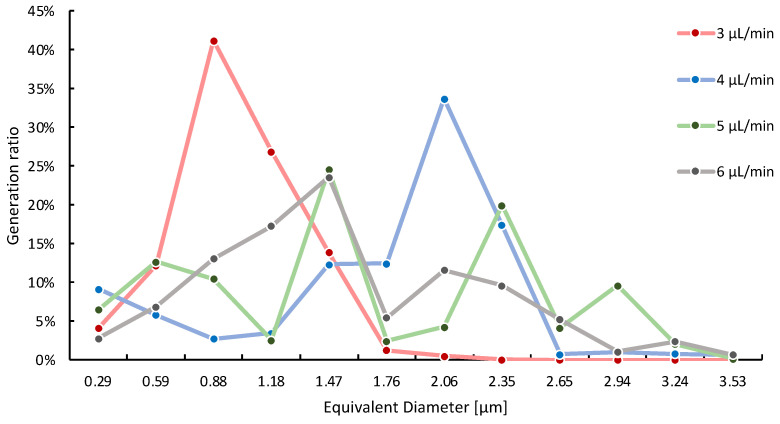
Relationship between the diameter of the generated S-MDs and continuous flow velocity.

Figure 4 shows the relationship between flow rate and droplet size distribution. The size of S-MD droplets was determined by the semantic segmentation of high-speed video frames. When the continuous flow rate is 3 μL/min, the generated droplets exhibit the most stable size distribution. Upon increasing the flow rate to 3, 4, 5, and 6 μL/min for continuous flow, the percentage of S-MD droplets with diameters below 2 μm is 99.47%, 45.83%, 59.08%, and 68.95%, respectively. Furthermore, approximately 100,000 S-MDs were generated per second at a flow rate of 3 μL/min.

**Figure 5:**
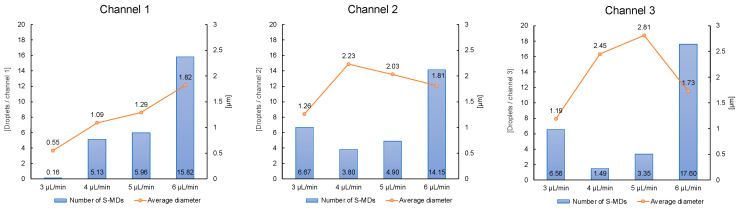
Variation in S-MD features with flow velocity.

In addition, the number and average diameter size of the droplets produced in the three consecutive channels at the central position was counted, as shown in Figure 5. By comparison, we can analyze that the higher the flow rate of the continuous phase, the higher the number of S-MD droplets generated, and the size of the generated S-MD is relatively stable (the average size is controlled below 2 μm). At a flow rate of 6 μL/min, droplets with a diameter of 2 μm were efficiently generated, and at a flow rate of 3 μL/min, droplets with a diameter of 1 μm were efficiently generated.

Consequently, we conclude that within the proposed flow pathway structure in this experiment, higher continuous flow rates result in a greater number of generated S-MD droplets and larger droplet diameters. The results of this fluid experiment suggest that it is possible to control the number and size of the S-MDs generated by changing the flow rate of the continuous phase.

## 3. Experimental Section

### 3.1. Fluid Simulation

Methanol is highly wettable for the PDMS wall, so a methanol stream cannot move apart from the wall to form droplets. In this experiment, a thin CYTOP layer was coated on the internal microfluidic device wall since methanol is less wettable for the CYTOP layer for droplet generation.

In order to quantify the effect of wettability, three-dimensional computational fluid dynamics (CFD) simulations were performed to evaluate droplet formation in a T-junction channel surface. The mass continuity and Navier–Stokes equations for incompressible fluids with constant density and the viscosity of the methanol and silicone oil described the momentum field. The set of questions was solved with commercial CFD software Star-CCML version 2023.6. The width and height of the T-junction were 50 μm. The wettability of the methanol in silicone oil for the PDMS wall was investigated as a function of the contact angle.

### 3.2. Device Design and Fabrication

**Figure 6:**
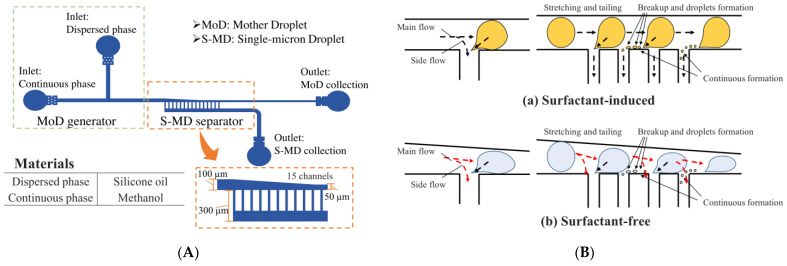
Schematic diagram and dimensions of the device. (**A**) Device design. (**B**) Comparison of the flow paths with and without added surfactant.

The design of the device used in this study is illustrated in Figure 6A). The device consists of two components: a mother droplet (MoD) generator and a single-micron droplet (S-MD) separator. The basic structure of the fluidic device is an adaptation of the device design we developed in 2021 [31] for methanol.

The MoD generator has two inlets for the dispersed and continuous phases arranged to form a T-shaped channel. The tapered flow channel in the S-MD separator allows the droplet to be easily deformed by the external forces from the flow channel.

As shown in Figure 6B), when the MoD enters the separation section from the main channel, tailing occurs due to the forces from the flow towards the main channel and the side channel. Figure 6(Ba) shows the S-MD generation device developed in 2021. This design required a surfactant even in methanol. On the other hand, the device developed in this research eliminates the need for a surfactant by tapering the channel width (Figure 6(Bb)).

Two outlets for MoD and S-MD collection were provided at the end and side of the device, respectively. The overall depth of the device was 30 µm. The width of the main channel was 100 µm, gradually narrowing to 50 µm by the end of the side channel region. Fifteen side channels, with a width of 25 µm and a length of 200 µm, blanched from the main channel spaced 75 µm apart. Methanol has a lower density and viscosity than water, so it tends to become unstable in the flow path, but by gradually constricting the main channel, the stable control of MoDs was achieved.

**Figure 7:**
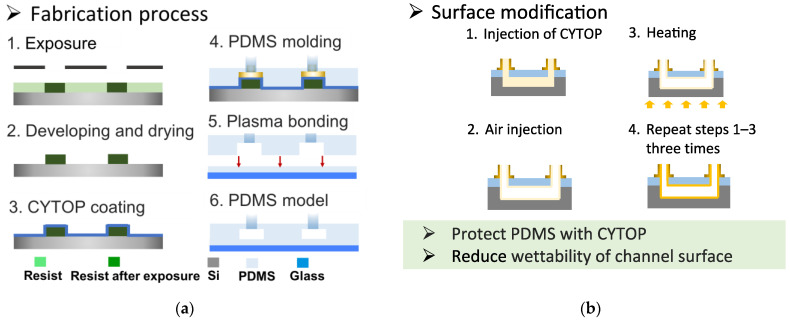
Flow charts showing the (**a**) device fabrication and (**b**) surface modification processes.

In this study, PDMS flow channel devices were fabricated by soft lithography followed by the surface treatment of the channels. The detailed process is summarized in Figure 7a. First, a mold was prepared on a silicon substrate by SU-8 (SU-8 3050, Kayaku Advanced Materials, Westborough, MA, USA) photolithography. This mold was then used to transfer the channel structure onto the PDMS. Next, the transferred PDMS was thermally cured, and the device was obtained by plasma bonding on a glass substrate with a PDMS thin film.

In addition, the channel wall surfaces were modified with CYTOP, which is chemically resistant to organic fluids and highly transparent, such that it did not interfere with fluid observations. Furthermore, CYTOP repels both water and oil, enabling the use of aqueous and organic dispersed phases. The surface modification was conducted as shown in Figure 7(b1–b4). 1. Introduce the CYTOP from the inlet of the microfluidic device. 2. Introduced air from the inlet to exhaust unnecessary CYTOPs. 3. Bake on a hot plate at 180 degrees for 60 min. 4. Repeat steps 1–3 three times.

### 3.3. Fluid Experiments

**Figure 8:**
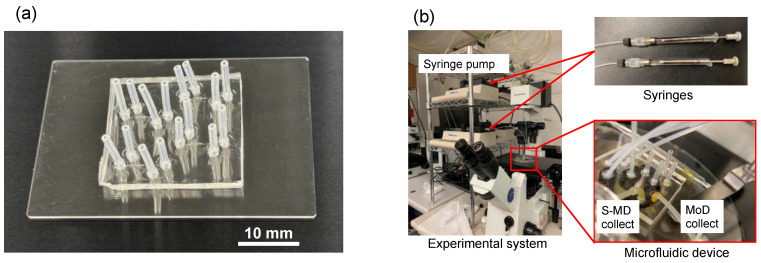
Microfluidic device appearance and experimental system. (**a**) PDMS microfluidic device. (**b**) Fluid experiment setup.

Figure 8 shows the appearance of the microfluidic device and the experimental system. Figure 8a is an external photograph of a PDMS microfluidic device. Four S-MD generation devices are fabricated on one chip, allowing efficient fluid experiments. Furthermore, since this PDMS device is coated with CYTOP, it is difficult for dirt to build up inside the flow path, so it can be used for repeated experiments.

Figure 8b shows the fluid experiment system. Silicone oil and methanol were used as the continuous and dispersed fluids, respectively. It is noteworthy that neither of these solutions contains any surfactant. The liquids were injected using syringes (1750CX, Hamilton, Reno, NV, USA) through PTFE tubing (i.d. 500 μm) or PVC tubing (i.d. 1000 μm), and syringe pumps (KDS, KD Scientific Inc., Holliston, MA, USA) were utilized to control the flow rate for the methanol and oil. In addition, we used an optical microscope and a high-speed video camera to observe the flow changes inside the flow path and record images and videos on a computer.

## 4. Conclusions

This study successfully developed a PDMS-based fluid device for separating S-MD with a diameter below 3 μm. Fluid experiments were carried out at flow rates of 3, 4, 5, and 6 μL/min for continuous flow, and at 1.5 μL/min for fixed dispersed flow. Single-micron droplets were stably generated under all conditions. These findings enhance our understanding of the relationship between flow rates and droplet generation for determining suitable conditions for studying the chemical reactions related to organic synthesis and provide valuable insights for further research and applications in related fields. Our study employed advanced techniques and methods, utilizing high-speed video frames and semantic segmentation to provide accurate and reliable data on droplet size and quantity. Furthermore, the incompatibility between PDMS and organic substances has always been a difficult problem to overcome. We successfully solved this problem by surface treatment using CYTOP, which extended the experimental time for organic liquids. The S-MD separation experiment was performed through the flow structure without the addition of any surfactant, creating a pure environment for the study of organic synthesis and biotechnology. Through this study, we successfully separated organic S-MDs, providing a platform for biological experiments and the production of new substances. We believe that this technology will have a positive impact on research in the fields of organic synthesis and biotechnology and provide new ideas and methods for the development of PDMS-based droplet separation systems.

## Data Availability

The data presented in this study are available in article.
